# Msb2 Shedding Protects *Candida albicans* against Antimicrobial Peptides

**DOI:** 10.1371/journal.ppat.1002501

**Published:** 2012-02-02

**Authors:** Eva Szafranski-Schneider, Marc Swidergall, Fabien Cottier, Denis Tielker, Elvira Román, Jesus Pla, Joachim F. Ernst

**Affiliations:** 1 Department Biologie, Molekulare Mykologie, Heinrich-Heine-Universität, Düsseldorf, Germany; 2 Departamento de Microbiología II, Facultad de Farmacia, Universidad Complutense, Madrid, Spain; University of Toronto, Canada

## Abstract

Msb2 is a sensor protein in the plasma membrane of fungi. In the human fungal pathogen *C. albicans* Msb2 signals via the Cek1 MAP kinase pathway to maintain cell wall integrity and allow filamentous growth. Msb2 doubly epitope-tagged in its large extracellular and small cytoplasmic domain was efficiently cleaved during liquid and surface growth and the extracellular domain was almost quantitatively released into the growth medium. Msb2 cleavage was independent of proteases Sap9, Sap10 and Kex2. Secreted Msb2 was highly *O*-glycosylated by protein mannosyltransferases including Pmt1 resulting in an apparent molecular mass of >400 kDa. Deletion analyses revealed that the transmembrane region is required for Msb2 function, while the large N-terminal and the small cytoplasmic region function to downregulate Msb2 signaling or, respectively, allow its induction by tunicamycin. Purified extracellular Msb2 domain protected fungal and bacterial cells effectively from antimicrobial peptides (AMPs) histatin-5 and LL-37. AMP inactivation was not due to degradation but depended on the quantity and length of the Msb2 glycofragment. *C. albicans msb2* mutants were supersensitive to LL-37 but not histatin-5, suggesting that secreted rather than cell-associated Msb2 determines AMP protection. Thus, in addition to its sensor function Msb2 has a second activity because shedding of its glycofragment generates AMP quorum resistance.

## Author Summary

Microbial pathogens are attacked by antimicrobial peptides (AMPs) produced by the human host. AMPs kill pathogens and recruit immune cells to the site of infection. In defense, the human fungal pathogen *Candida albicans* continuously cleaves and secretes a glycoprotein fragment of the surface protein Msb2, which protects against AMPs. The results suggest that shed Msb2 allows fungal colonies to persist and avoid inflammatory responses caused by AMPs. Msb2 shedding and its additional role in stabilizing the fungal cell wall may be considered as novel diagnostic tools and targets for antifungal action.

## Introduction

Crosstalk between pathogens and the human host determines the outcome of microbial colonization and disease [1]. Pathogen-host communication occurs between cells and secreted proteins of both organisms. Surface structures of the important human fungal pathogen *Candida albicans* bind to dectin receptors on immune cells and trigger responses inhibiting fungal proliferation including the production of antimicrobial peptides (AMPs) and reactive oxygen species (ROS) (for a review, see [2], [3]. In addition, binding to immunoglobulins and complement factors by the fungal pathogen facilitate its phagocytosis and killing (for a review, see [4]). Conversely, *C. albicans* partially overcomes host defenses by secreting hydrolytic enzymes and proteins that block the complement system (for a review, see [4], [5]). Furthermore, by switching its growth from a yeast to a hyphal growth form *C. albicans* is able to evade immune cells and to penetrate into host niches less accessible to the immune system.

Survival of fungal pathogens in the human host requires that their cell surfaces are intact. Defects in the cell wall of *C. albicans* that occur under immune attack or by treatment with antifungals are sensed and activate compensatory activities [6]. Reduced glucan content leads to the activation of the protein kinase C (PKC) pathway that includes the Mkc1 MAPK module, which activates the glucan synthase activity and stimulates the transcription of genes involved in glucan and chitin biosynthesis [7], [8]. In addition, defective *N-* or *O-*glycosylation activates the Cek1 MAPK module and recent results indicate that *PMT* genes encoding protein-*O*-mannosyltransferases are downstream regulatory targets [9], [10]. Sensing through this pathway is accomplished by the Msb2 and Sho1 cytoplasmic membrane proteins, which signal via the Cdc42 GTPase to Cek1. Intact *N*-glycosylation is detected by Msb2 and represses *PMT1* transcription, while defective *N*-glycosylation induces Cek1 phosphorylation and de-represses *PMT1* transcription [9], [10]. In a different mode of regulation, defective Pmt1-type *O*-glycosylation is sensed by Msb2, activates Cek1 and induces *PMT2* and *PMT4* expression. Induction of *PMT2/PMT4* genes by inhibition of Pmt1 and damage of β1,3-glucan also requires Msb2 and Cek1 suggesting that cell wall damage is reported to Cek1 via Msb2 [10]. This function of Msb2 is supported by its associated partner membrane protein Sho1 [9]. Defects in either Mkc1 or Cek1 pathways lead to defective hypha formation on some semi-solid media, supersensitivity against antifungals and other stressors and reduce the virulence of *C. albicans* [9], [11], [12].

Msb2 is a type I membrane protein containing a single transmembrane region that separates a large extracellular from a small cytoplasmic domain; this structure is conserved in several fungal species [13]–[16]. Msb2 in the yeast *Saccharomyces cerevisiae* has been shown to be continuously cleaved by the Yps1 yapsin protease, releasing the extracellular domain into the growth medium [17]. This property, coupled with the high level of *N-* and *O-*glycosylation of the extracellular domain has led to the concept that fungal Msb2 proteins represent functional analogs of the mammalian MUC1/2 signaling mucins, which by proteolytic cleavage generate highly hydrated mucous glycoprotein layers around cells and at the same time confer transcriptional regulation by the cleaved cytoplasmic domain [18]. In fungi, intertwining of Msb2 hydrated glycostructures with cell wall components may be related to the sensing function of Msb2. Cleavage of the ScMsb2 cytoplasmic domain has not been reported and its presence may be required for Cdc42 binding, which is an essential upstream element of the Kss1 MAPK pathway [13]. Here we report that the glycosylated extracellular domain of *C. albicans* Msb2 is released into the growth medium in considerable amounts and we show that the shed protein has the function to protect against AMPs produced by the host. In humans, the most prominent AMPs exhibiting strong antimicrobial and immunostimulatory activities are the histatins, which are produced by salivary glands and secreted into saliva and the cathelicidins and defensins, which are produced by neutrophils and macrophages (for a review, see [19]–[21]). The human cathelicidin LL-37 occurs on mucosal surfaces at a concentration of 2–5 µg/ml but its concentration rises to 1.5 mg/ml in acute inflammation [22]. Histatin-5 and LL-37 are cationic AMPs that damage the cytoplasmic membranes of *C. albicans* [23]–[25] and histatin-5 also attacks intracellular targets [26]. The combined findings of this study suggest that shed Msb2 is a glycoprotein that effectively protects *C. albicans* against killing by AMPs LL-37 and histatin-5, allowing *C. albicans to* evade immune reactions and to allow its persistence as a commensal.

## Results

### Construction and activity of epitope-tagged Msb2

**Figure 1:**
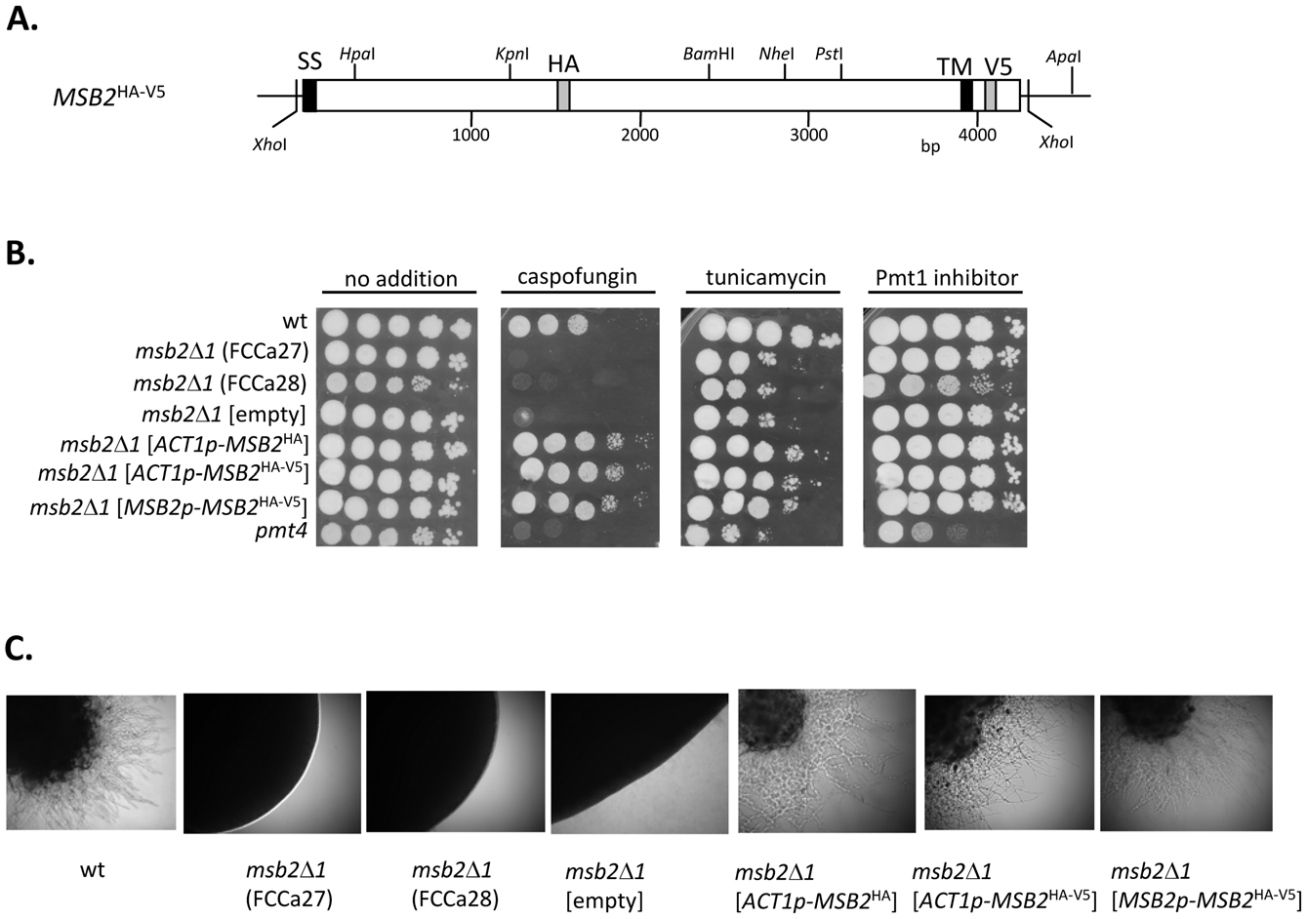
Structure and activity of epitope-tagged Msb2. **A.** Structure of *MSB2* alleles encoding Msb2 variants. The *MSB2* coding region with sequences encoding the signal sequence (SS), the transmembrane region (TM), HA- /V5-epitopes and relevant restriction sites used for the construction of *MSB2* variant alleles are shown. **B.** Tagged *MSB2* alleles confer inhibitor resistance. Sensitivity of strains to caspofungin (125 ng/ml), tunicamycin (2 µg/ml) and Pmt1 inhibitor (12 µM) was tested by a drop dilution test. **C.** Tagged *MSB2* alleles reconstitute formation of hyphae on YPM agar. Colonies of strains were photographed following growth for 2 d at 37°C. Strains CAF2-1 (wt), FCCa27 (*msb2*Δ*1 URA3*) and FCCa28 (*msb2*Δ*1 ura3*) were compared to FCCa28 transformants. Transformants contained empty plasmid pDS1044-1 (ESCa7; *msb2*Δ*1*[empty]), pES10 (ESCa8; *msb2*Δ*1*[*ACT1p*-*MSB2* ^HA^]) or pES11a (ESCa3; *msb2*Δ*1*[*ACT1p*-*MSB2* ^HA-V5^]) integrated in the *LEU2* locus. The tagged *MSB2* allele was also placed under transcriptional control of the authentic *MSB2* promoter by directing integration of *Hpa*I-cut pES11a into the *msb2*Δ*1* allele of FCCa28 (ESCa10; *msb2*Δ*1* [*MSB2p*-*MSB2* ^HA-V5)^]). Strain CAP4-2164 (*pmt4*) was used as a supersensitive control strain [27].

To immunologically detect Msb2 we constructed a strain producing a variant Msb2 protein carrying an HA-epitope within the large extracellular domain and in addition a V5-epitope in the middle of the short cytoplasmic domain (Figure 1 A). *MSB2* was expressed either under the control of the constitutive *ACT1* promoter when plasmid pES11a was integrated in the *LEU2* locus (strain ESCa3) or by the authentic *MSB2* promoter when pES11a was integrated in the partially deleted *msb2*Δ*1* allele of strain FCCa28 (strain ESCa10). The *msb2*Δ*1* allele encoding 406 N-terminal residues of Msb2 was found to be completely non-functional in all phenotypic assays (see below) and it was fully complemented in transformants containing pES11a integrated in both genomic loci; complementation efficiencies were equal between transformants carrying singly HA-tagged or doubly HA-V5-tagged Msb2 versions. Thus, while several *msb2*Δ*1* mutant strains were as supersensitive to caspofungin and tunicamycin as the *pmt4* control strain [10] complementation by the epitope-tagged versions of Msb2 restored normal resistance (Figure 1 B). While tunicamycin-supersensitivity indicates that *msb2*Δ*1* mutants require intact *N-*glycosylation for growth, *O-*mannosylation by Pmt1 appears not relevant since mutants grew normally in the presence of the Pmt1 inhibitor. The tagged versions of Msb2 were also fully active to reverse the hyphal growth defects of the *msb2*Δ*1* mutants [9] (Figure 1 C).

In addition, we constructed plasmid pES11c, which encodes the HA-tagged Msb2 variant carrying the V5 epitope at its C-terminal end (allele *MSB2* ^HA-V5 end^). The phenotypic results for pES11a- and pES11c-transformants were identical (data not shown).

### Secretion and processing of Msb2

**Figure 2:**
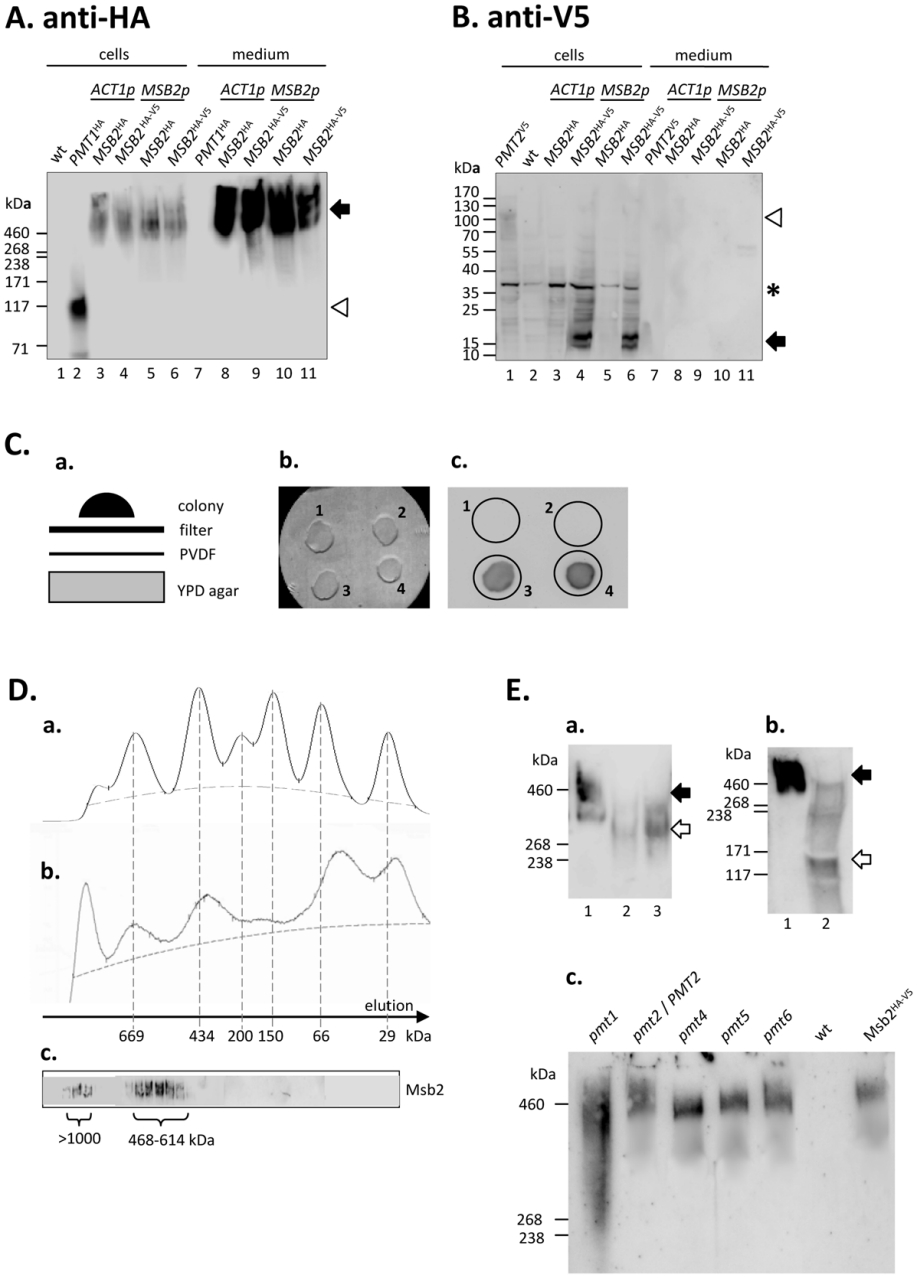
Secretion and processing of Msb2. *C. albicans* strains grown in YPD medium to OD_600_ = 6, centrifuged and cell extracts (50 µg protein derived from cells in 90 µl of medium) or medium (20 µl) were analyzed for epitope-tagged Msb2 protein. **A.** Immunoblot to detect HA-tagged Msb2. Proteins were separated by a 8% SDS-PAGE gel and immunoblots were reacted with rat anti-HA antibody. Strains tested included ESCa8 (*ACT1p*-*MSB2* ^HA^; lanes 3 and 8), ESCa3 (*ACT1p*-*MSB2* ^HA-V5^; lanes 4 and 9), ESCa9 (*MSB2p*-*MSB2* ^HA^; lanes 5 and 10) and ESCa10 (*MSB2p*-*MSB2* ^HA-V5^; lanes 6 and 11). Strains CAF2-1 (wt) and CIS23 (*PMT1* ^HA^) were used as negative and positive control strains, respectively. The migration of HA-tagged Msb2 and Pmt1 are indicated by the arrow and triangle, respectively. **B.** Immunoblot to detect V5-tagged Msb2. Proteins were separated by a 4–20% gradient SDS-PAGE gel and immunoblots were reacted with mouse monoclonal anti-V5 antibody. Identical strains and fractions as in (A) were tested. The migration of V5-tagged Msb2 and Pmt2 (strain CIS29) are indicated by the arrow and the triangle, respectively; a protein cross-reacting with the anti-V5 antibody is marked by the asterisk. **C.** Secretion of HA-tagged Msb2 protein during growth on agar. Cell suspensions were dropped on a membrane filter (pore diameter 0.45 µm) situated on a PVDF membrane, which had been placed on YPD agar (a). Colonies were allowed to grow for 15 h at 30°C (b). The membrane filter was removed and the PVDF membrane was probed by immunoblotting using rat anti-HA antibody (c). Strains tested were (1) CAF2-1 (wild-type), (2) CIS23 (*PMT1* ^HA^), (3) ESCa3 (*ACT1p*-*MSB2* ^HA-V5^) and ESCa10 (*MSB2p*-*MSB2* ^HA-V5^). **D.** Gel filtration chromatography of secreted Msb2. A Superdex 200 10/300 GL column was (a) calibrated using standard proteins of the indicated sizes (dotted lines) and (b) used to fractionate 500 µl of the medium of strain ESCa3 (Msb2^HA-V5^), which had been grown at 30°C in SD medium to OD_600_ = 10. The protein elution profiles were recorded by absorption at 280 nm. 200 µl fractions were collected and (c) tested by immunoblotting for the presence of HA-tagged Msb2. Fractions tested are placed at a position corresponding to the elution profile in b). **E.** Glycosylation of secreted Msb2. (a) Growth medium of strain ESCa3 (Msb2^HA-V5^) was not treated (1) or treated with β-elimination reagent mixture over night (2,3); the sample in lane 3 was heated to 80°C before reagent addition in an attempt to increase deglycosylation. (b) The medium was not treated (1) or treated with TFMS (2). Samples were tested by immunoblotting as in A. The migration of glyosylated and deglycosylated Msb2* are indicated by the filled and open arrows, respectively. (c) Msb2 secreted by *pmt* mutants defective in protein-*O*-mannosyltransferases carrying carried pES11a (Msb2^HA-V5^). Strains included ESca18 (*pmt1*), ESCa19 (*PMT2/pmt2*), ESCa20 (*pmt4*), ESCa21 (*pmt5*) and ESCa22 (*pmt6*) and were tested by immunblotting as in A.

Release of a Msb2 subfragment into the growth medium has been observed in *S. cerevisiae* and other fungi [13]–[16]. When we examined cells and growth medium of *C. albicans* transformants producing tagged Msb2 by immunoblotting we discovered that the majority of HA-carrying Msb2 was present in the medium and migrated as a diffuse band of >460 kDa (Figure 2 A). No significant difference regarding the amount of immunoreactive protein was detected in strains either transcribing *MSB2* from the *ACT1* or *MSB2* promoters (compare lanes 3 and 5) suggesting that both promoters are of comparable strength. As expected, the tagged ER-membrane protein Pmt1^HA^ was associated only with cells (lane 2). In contrast to HA immunodetection the V5-tagged Msb2 protein was found exclusively in association with cells and not in the medium, similar to the Pmt2^V5^ control protein (Figure 2 B). The V5-tagged Msb2 protein migrated as a doublet of about 15 kDa and thus corresponded in size to the cytoplasmic domain of Msb2. Thus, it appears that during growth in liquid culture the Msb2 full-length protein is mostly cleaved proteolytically into the large extracellular (HA-tagged) and the small cytoplasmic (V5-tagged) subfragments. Importantly, release of the Msb2^HA^ fragment was almost quantitative during growth in complex YPD growth medium and was not altered significantly in YEPG medium containing galactose as in *S. cerevisiae* [17] or during hypha formation in YP medium containing 10% serum (data not shown). The released extracellular fragment or Msb2 will now be referred to as Msb2*.

To examine if Msb2* secretion would also occur during growth on a semisolid agar surface we used a double sandwich system consisting of a PVDF membrane used for immunoblotting topped by a membrane filter precluding the passage of cells, which were both placed on YPD agar (Figure 2 C, a). Cells grew on the membrane filter (Figure 2 C, b) and immunoanalysis of the PVDF filter detected HA-proteins only released from cells producing Msb2* (Figure 2 C, c 3, 4) but not from cell producing tagged Pmt1^HA^ protein. This result indicates that the extracellular Msb2 fragment is also detected in surface growth of *C. albicans*.

**Figure 3:**
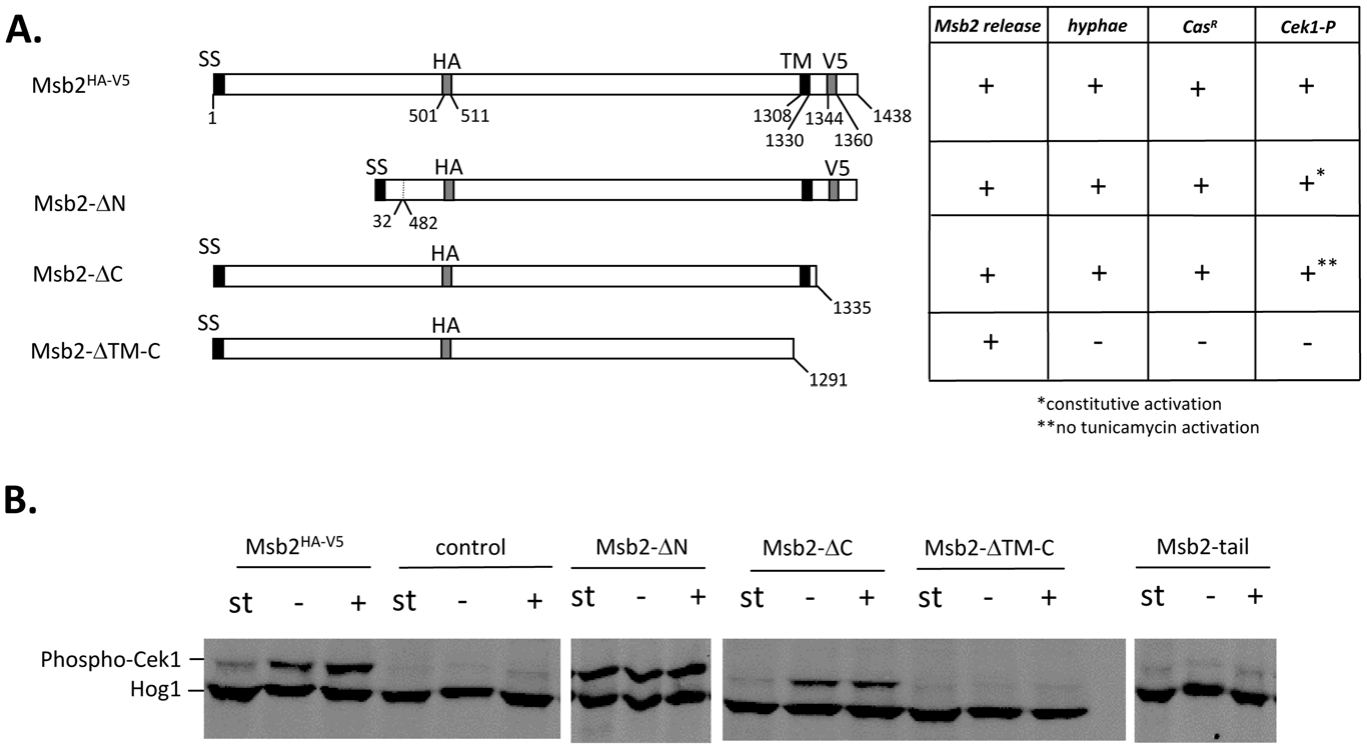
Activity of Msb2 variants. **A.** Structure of Msb2 protein variants. The positions of signal sequence (SS), transmembrane region (TM) and HA- and V5-epitope tags are indicated. Plasmids encoding variants were chromosomally integrated into strain FCCa28, which produces the inactive Msb2-Δ1 variant by the *msb2*Δ*1* allele. Resulting transformants (encoded variants) were strains ESCa3 (Msb2^HA-V5^), ESCa25 (Msb2-ΔN), ESCa38 (Msb2-ΔC) and ESCa39 (Msb2-ΔTM-C). Corresponding phenotypes are summarized in the table and are presented in Figure S1. +, wild-type phenotype; −, *msb2* mutant phenotype with regard to Msb2* release, hypha formation, caspofungin resistance (Cas^R^) and Cek1 phosphorylation (Cek1-P). **B.** Cek1 activation by strains producing variant Msb2 proteins. Cells were grown to stationary phase (st), diluted in fresh YPD medium, grown to OD_600_ = 0.8 at 37°C and incubated further for 1 h in the presence (+) or absence (−) of tunicamycin (2 µg/ml). Cells in stationary phase (st) and after 1 h incubation were harvested and assayed for the activation of MAPK Cek1 by immunoblottings; the Hog1 MAPK protein signal was used as the loading control. Strains as in A., in addition strains ESCa37 encoding the Msb2-tail variant and strain ESCa7 carrying an empty vector (control) were tested.

**Figure 4:**
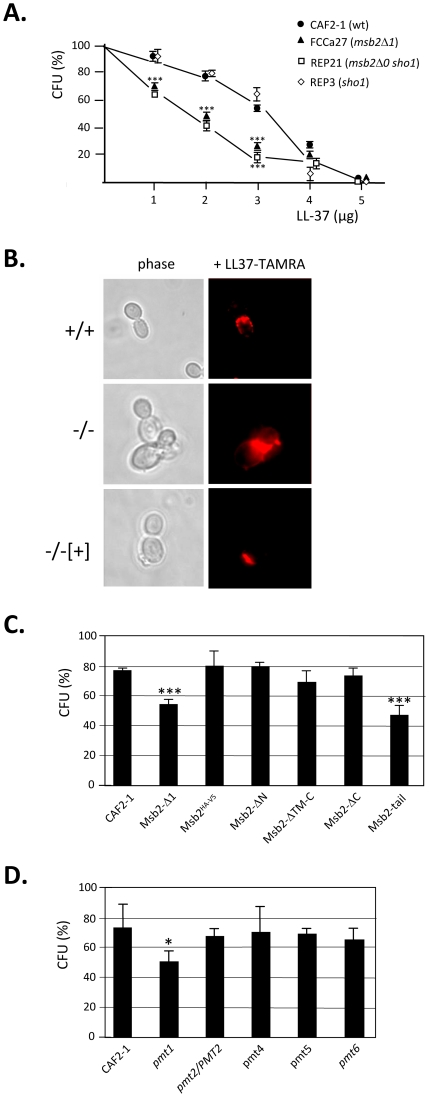
Msb2 synthesis protects *C. albicans* against LL-37. **A.** Basal LL-37-resistance of *C. albicans* depends on Msb2. The indicated strains were incubated with different LL-37 amounts for 1.5 h at 37°C before plating of cells to determine colony-forming units (CFUs). Standard deviations of triplicate measurements are indicated; statistical differences of mutant versus control strain cfu values were evaluated by a t-test. **B.** Staining of *C. albicans* by TAMRA-labelled LL-37. 50 µl of cells were resuspended in PBS were incubated for 5 min with 5 µg LL-37-TAMRA before visualization using phase contrast and fluorescence microscopy. CAF2-1. +/+; FCCa27, −/−; ESCa3, −/−[+]. **C, D.** LL-37 sensitivity of *C. albicans* strains producing Msb2 variants (C) and of *pmt* mutants producing undeleted Msb2 (D). Transformants producing variant Msb2 proteins are described in Figures 2 and 3. Strain suspensions (5 µl) were co-incubated with 2 µg LL-37 for 1.5 h before CFU determination. Means and standard deviations of triplicate assays are shown. Statistical significance using a t-test is indicated by * (p<0.05), ** (p<0.01) and *** (p<0.001).

Considering the possibility that Msb2 is cleaved immediately upstream of the transmembrane region it was expected that Msb2* has an approximate molecular mass of 131 kDa but the heterogeneity and apparent molecular mass in immunoblotting (Figure 2 A) suggested extensive glycosylation. To estimate its molecular mass more accurately we carried out fractionation of culture fluid containing Msb2* by gel filtration, using a column previously calibrated with standard proteins (Figure 2 D, a, b). Fractions eluted from the column were examined by immunodetection and yielded a major peak from 468–614 kDa (Figure 2 D, c) in agreement with the above immunoblotting results. A minor peak in the void volume, presumably representing aggregated Msb2* of >1000 kDa, was also detected. Since this result suggested that glycosylation contributed equally to the mass of Msb2* as its protein content we attempted to clarify the type of protein glycosylation. Extensive treatment of the growth medium (and of purified Msb2*, see below) with PNGase F did not result in a significant alteration of its apparent molecular mass (data not shown), while β-elimination led to a mass reduction to about 300 kDa (Figure 2 E, a) indicating that Msb2* is significantly *O*-but not *N*-glycosylated. On the other hand, complete chemical deglycosylation by trifluoromethanesulfonic acid (TFMS) reduced the mass of Msb2* to about 117–130 kDa (Figure 2 E, b) consistent with the proteolytic cleavage of the Msb2 precursor protein immediately upstream of the transmembrane region (expected molecular mass of unmodified 1291 residue fragment is 130 kDa). It is yet unclear if the different deglycosylation results obtained for β-elimination and TFMS treatments is due to residual *O*-glycosylation not removable by β-elimination, by residual *N*-glycosylation, which is not removed by PNGase F or by yet unknown modifications. However, because clear evidence for *O-*glycosylation of secreted Msb2 was obtained we produced epitope-tagged Msb2 in *C. albicans* mutants lacking each of the 5 isoforms of protein-*O*-mannosyltransferases. Immunoanalysis of secreted Msb2* showed faster electrophoretic mobility in the *pmt1* mutant, while in the *pmt4*, *pmt5* and *pmt6* homozygous mutants no difference to the control strain was detected (Figure 2 E, c). We conclude that Pmt1 is at least partially involved in Msb2 *O*-glycosylation, although the contribution of Pmt2 (only testable in a heterozygous *PMT2*/*pmt2* strain since it is essential for growth [27]) cannot be excluded. Compensatory upregulation of other Pmt isoforms in a *pmt1* mutant [10], [28] may also account for remaining Msb2 *O-*glycosylation, which showed a very broad mobility distribution corresponding to apparent molecular masses from 240–480 kDa.

It has been reported that in *S. cerevisiae* the yapsin-type protease Yps1 is responsible for cleavage and secretion of Msb2 [17]. In *C. albicans* the closest homolog to Yps1 is Sap9 (21.9% identity), while Sap10 is also structurally similar because it is GPI-anchored in the cytoplasmic membrane [29]. When we expressed the tagged *MSB2* ^HA-V5^ allele in the *sap9* mutant (ESCa33), the *sap10* mutant (ESCa34) or the *sap9 sap10* double mutant (ESCa35) we did not observe any difference in amounts and molecular masses of Msb2* (data not shown). We also observed normal secretion of Msb2 in a mutant (ESCa36) lacking the furin-type and Golgi-resident Kex2 serine endoproteinase, which in *S. cerevisiae* is required for cleavage and shedding of the Flo11 protein [30]. Furthermore, we repeatedly added high concentrations (15 µg/ml) of the aspartyl protease inhibitor pepstatin, of the metalloprotease inhibitor amastatin (15 µg/ml) or of a commercial mix of inhibitors for serine- and cysteine proteases (complete mini tablets; Roche) to growing cultures of ESCa3 but we did not find any effect on Msb2* release (data not shown). We conclude that the processing mechanism of Msb2 in *C. albicans* requires an as yet unidentified protease and that Sap9, Sap10 and Kex2 proteases are not involved.

### Structure-function relationship of Msb2

We constructed several *C. albicans* strains producing deleted Msb2 variants under the control of the *ACT1* promoter in a *msb2* mutant background and tested Msb2-dependent phenotypes including secretion of Msb2, hypha formation and resistance to caspofungin; furthermore, the ability of variants to activate the Cek1 MAP kinase was examined. The results are summarized in Figure 3 A and presented in Figure 3 B and Figure S1.

Two major deletion variants either lacking 449 residues of the extracellular domain (Msb2-ΔN) or lacking the complete cytoplasmic tail of 103 residues (Msb2-ΔC) were fully able to complement all *msb2* mutant phenotypes. In contrast, strains only producing the N-terminal region of Msb2 up to the transmembrane region (variant Msb2-ΔTM-C) or solely the 108 cytoplasmic variant Msb2 tail residues were as defective for Msb2 phenotypes as mutants REP18 carrying a complete deletion of the *MSB2* ORF or strain FCCa27 only producing N-terminal residues 1–406 of Msb2 (Msb2-Δ1). Inactivity of the Msb2-ΔTM-C variant was not caused by lack of protein biosynthesis since amounts of Msb2* released into the medium were comparable for all HA-tagged variants (Figure S1). However, with regard to the activation of Cek1 a particular phenotype of these deletion variants was observed. The wild-type strain ESCa3 showed low levels of phosphorylation in stationary phase and phosphorylation was increased during logarithmic growth, which was stimulated further in the presence of tunicamycin [9] (Figure 3 B). In contrast, strain ESCa25 producing the Msb2-ΔN variant activated Cek1 not only in stationary phase but also in the absence of tunicamycin to high levels. In addition, strain ESCa38 carrying the Msb2-ΔC variant was impaired in its ability to activate Cek1 in response to tunicamycin. Strains producing the Msb2-ΔTM-C and the Msb2-tail were completely unable to activate Cek1 phosphorylation. Thus, it appears that the Msb2 N-terminal, transmembrane and cytoplasmic domains region convey different functions in Cek1 phosphorylation.

### *C. albicans msb2* are supersensitive to LL-37

*C. albicans* ESCa3 expressing *ACT1p-MSB2* ^HA-V5^ released considerable amounts of the Msb2* glycoprotein into the complex YPD growth medium, amounting to 76 µg/ml and 150 µg/ml in logarithmic growth (OD_600_ = 1) and in stationary phase (OD_600_ = 6). Msb2* was quantitated immunologically by a dot-blot procedure, because its high glycosylation status prevented quantitation by standard methods. We considered that this glycoprotein could contribute to defense against immunological responses of the human host, in particular to the attack by AMPs [20]. To verify this concept we first tested if the presence of Msb2 would contribute to basal levels of AMP resistance of *C. albicans*. Wild-type strains were significantly more LL-37-resistant than *msb2* mutants (Figure 4 A). Sensitivity of a *msb2 sho1* double mutant was only slightly increased compared to a *msb2*Δ*1* single mutant and a *sho1* single mutant showed wild-type resistance indicating that Msb2 but not Sho1 mediates LL-37 resistance. The increased LL-37 sensitivity of *msb2* mutant strains versus a wild-type strain was also correlated with increased fluorescent staining of mutant cells [26], [27] by TAMRA-labelled LL-37 (Figure 4 B). We also observed that in the presence of LL-37 the *msb2* mutant tended to aggregate more readily than wild-type cells [31].

We next tested the LL-37 sensitivity of the above series of transformants producing truncated Msb2 variants. Interestingly, while the transformant only synthesizing the C-terminal tail of Msb2 was as sensitive as the *msb2*Δ*1* mutant all other transformants showed wild-type sensitivity (Figure 4 C). Even the transformant producing Msb2 deleted for its transmembrane region and C-tail was not supersensitive, although as described above this Msb2 variant was inactive in complementing *msb2* mutant phenotypes (Figure 3). It was concluded that the basal resistance of *C. albicans* to LL-37 depended on the secreted extracellular domain of Msb2 but its N-terminal domain was not required for this action. Since full-length and N-terminally deleted Msb2* are *O-*glycosylated to a large part by Pmt1 (Figure 2) transformants were constructed producing doubly tagged Msb2 in *C. albicans* strains defective in each of the 5 Pmt proteins (a heterozygous strain was used in case of *PMT2* because of its essentiality for growth). Among these transformants only the *pmt1* mutant was LL-37 supersensitive supporting the notion that Pmt1-directed *O*-glycosylation of Msb2* is required to provide resistance to LL-37.

In conclusion, these results suggest that the secreted extracellular Msb2* domain is required for LL-37 basal resistance of *C. albicans*.

### Secreted Msb2 protects against AMPs

**Figure 5:**
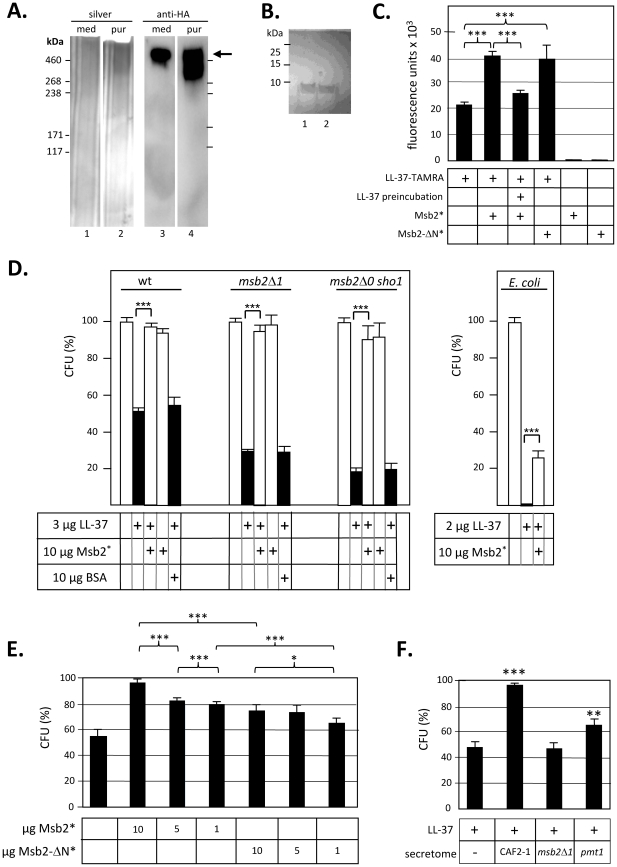
Msb2*-mediates protection of *C. albicans* and *E. coli* against LL-37. **A.** Purification of Msb2*. Msb2* in culture medium of strain ESCa3 (lanes 1, 3) was affinity-purified using an anti-HA column (lanes 2, 4) and samples were separated by SDS-PAGE (4–20% acrylamide gel). For silver staining 50 µl of medium/purified (med/pur) fractions (lanes 1, 2) and for immunoblotting using an anti-HA antibody 15 µl of medium/purified fractions (lanes 3, 4) was analyzed. **B.** Msb2 does not degrade LL-37. 3 µg of LL-37 were co-incubated without (lane 1) or with 10 µg of Msb2* (lane 2) for 1.5 h at 37°C. Samples were separated on a 18% SDS-PAGE gel; the migration of standard proteins is indicated. **C.** Immobilized Msb2* binds LL-37. 10 µg Msb2* or Msb2-ΔN* were allowed to attach to each well of polystyrene microtiter plates over night at 4°C. Wells were washed with PBST and unspecific binding sites were removed by incubation with skim milk solution. 5 µl (5 µg) of TAMRA-labelled LL-37 was allowed to bind for 1 h, wells were washed and TAMRA emission was recorded at 590 nm. As a control, coated wells were incubated first with 3 µg of unlabelled LL-37 for 1 h before addition of LL-37-TAMRA. **D.** Msb2*-mediated protection of *C. albicans* and *E. coli* against LL-37. Strain suspensions (5 µl) were co-incubated with LL-37 in the absence or presence of 10 µg Msb2* or 10 µg of its deleted variant Msb2*-ΔN for 1.5 h before determination of viable cell counts (CFUs). As a control, 10 µg BSA was used to replace Msb2*. *C. albicans* strains CAF2-1 (wt), FCCa27 (*msb2*Δ*1*), REP21 (*msb2*Δ*0 sho1*) and *E. coli* DH5αF′ were tested; means and standard deviations of triplicate assays are shown. **E.** Msb2* concentration dependence. *C. albicans* strain CAF2-1 was incubated with 3 µg LL-37 in the absence and presence of the indicated amounts of purified Msb2* and the deleted Msb2*-ΔN variant. In addition, the activity of HA peptides used for affinity purification of Msb2* was tested. **F.** Protection of *C. albicans* against LL-37 by medium proteins (secretome). 3 µg LL-37 was added to cells of strain CAF2-1 (5 µl; OD_600_ = 0.3) in the absence or presence of culture medium (17 µl) of *C. albicans* strains grown to stationary phase. Following incubation for 1.5 h at 37°C cell viability (CFU) was tested. Secretome of control strain CAF2-1, FCCa27 (*msb2*Δ*1*) and SPCa2 (*pmt1*) was used. Means and standard deviations of triplicate assays are shown. Statistical significance was evaluated using a t-test (*, p<0.05; **, p<0.01; ***; p<0.001).

Several mechanisms are possible to explain the requirements of Msb2 (and Sho1) for LL-37 resistance and one mechanism is inactivation of LL-37 by the secreted Msb2^*^. To verify this concept we first purified Msb2* fragment from the growth medium by affinity chromatography using anti-HA antibody and verified that the purified material consisted solely of the heterogeneous >460 kDa protein by silver staining and immunoblotting (Figure 5 A). Next we asked if the purified Msb2* would proteolytically attack cathelicidin LL-37. Msb2^*^ and AMPs were co-incubated and then assayed AMPs on a 18% SDS-PAGE gel (which excludes Msb2^*^). Msb2^*^ co-incubation did not diminish amounts of LL-37 and no degradation products were observed (Figure 5 B) even if a 22.5% SDS-PAGE gel was used (data not shown). Furthermore, long term incubations (16 h) of Msb2* preparations with substrates of a protease detection kit able to detect a wide variety of protease did not detect any protease activity (data not shown). Therefore, it was concluded that Msb2* preparations had no general proteolytic activity. In additional pre-tests we bound Msb2* (or Msb2-ΔN*) to wells of microtiter dishes and checked if TAMRA-labelled LL-37 would absorb to these wells. Msb2* coating did indeed stimulate binding of LL-37-TAMRA significantly, while preincubation with unlabelled LL-37 reduced subsequent binding (Figure 5 C). This result indicates that LL-37 has a specific binding site on Msb2*.

To test a potential function of Msb2* in AMP protection we set up an AMP activity assay, in which we treated *C. albicans* for 1.5 h with AMPs in the absence or presence of purified Msb2^*^ and then assessed fungal viability by determination of colony-forming units (CFU). The results show that added Msb2^*^ rescued *C. albicans* from LL-37 killing, which was obvious for the wild-type strain and even more significant for *msb2* and *msb2 sho1* mutants; even an *E.coli* strain was protected against LL-37 by Msb2* (Figure 5 D). Interestingly, even the shortened Msb2*-ΔN fragment secreted and purified from strain ESCa25 was able to provide protection, although a concentration dependence of its activity revealed that it is slightly less active in AMP inactivation compared to the full-length Msb2^*^ protein (Figure 5 E). AMP inactivating activity was also detected by merely using medium (secretome) of a *C. albicans* wild-type strain (CAF2-1) for co-incubation with LL-37 (Figure 5 F). As expected, medium of the *msb2*Δ*1* strain (FCCa27) had no protective effect, while medium of the *pmt1* mutant (SPCa2) had reduced inactivating activity.

These findings demonstrate that the extracellular Msb2 domain has an additional function in *C. albicans* biology, e. g. in LL-37 defense, which is different from its roles in cell wall integrity and filamentation.

### Secreted Msb2 protects *C. albicans* against histatin-5

**Figure 6:**
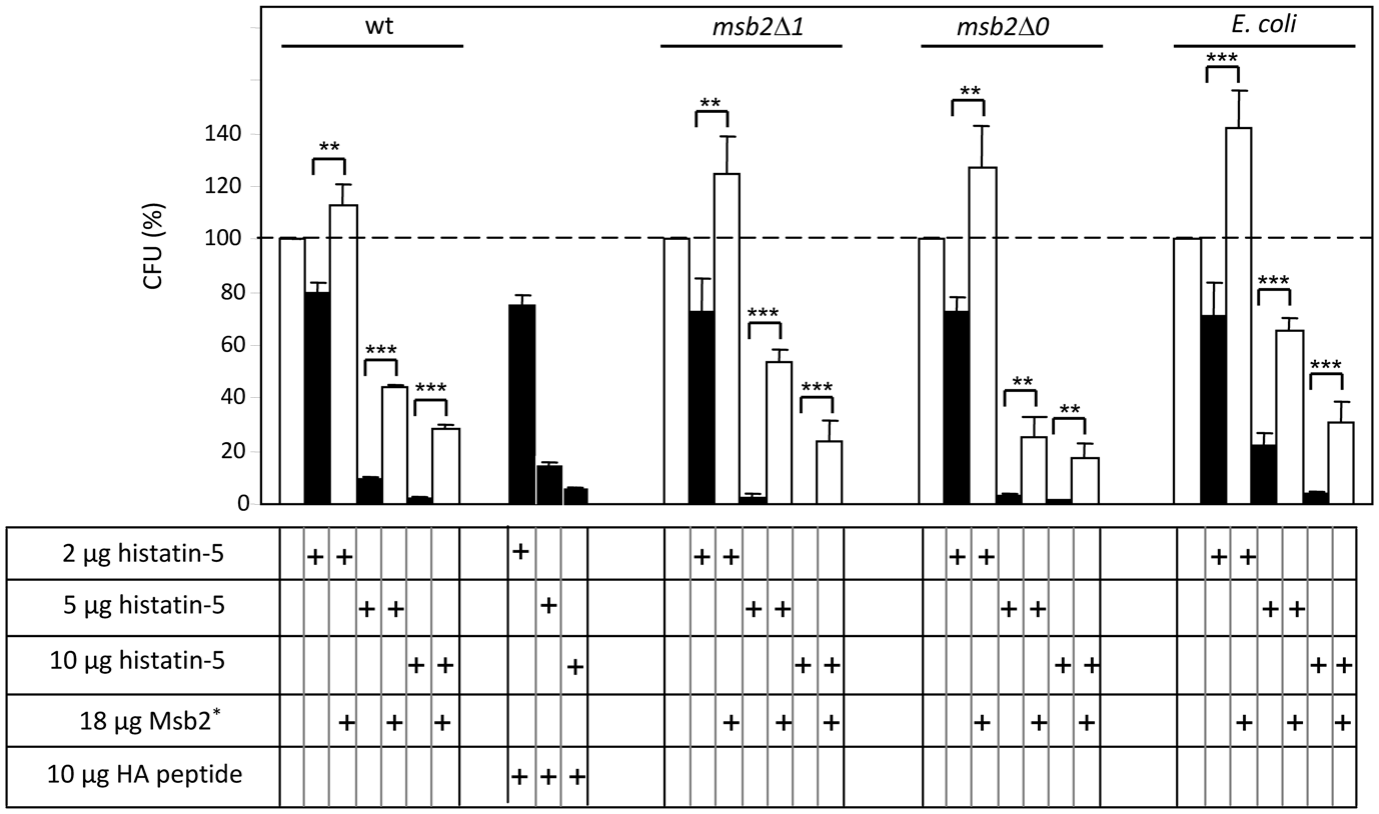
Msb2*-mediates protection of *C. albicans* and *E. coli* against histatin-5. *C. albicans* strains CAF2-1 (wt), FCCa27 (*msb2*Δ*1*), REP18 (*msb2*Δ*0*) and *E. coli* DH5αF′ were allowed to react with the indicated amounts of histatin-5 for 1.5 h at 37°C, in the absence or presence of the affinity-purified secreted Msb2* protein. Colony-forming units were determined on YPD (*C. albicans* strains) or on LB medium (*E. coli*).

*C. albicans* is known to be sensitive to low levels of histatin-5 [23]–[26], [32], [33]. We considered the possibility that higher Msb2^*^ levels occurring in the vicinity of *C. albicans* colonies in the human host could protect against histatin-5 as we had found for LL-37. Although we did not observe a significant higher sensitivity to histatin-5 in *msb2* mutants (as for LL-37) we found that added purified Msb2^*^ did indeed protect *C. albicans* strains significantly against histatin-5 (Figure 6). As expected, HA peptide used for elution of Msb2* from the anti-HA antibody in affinity chromatography did not provide protection. The protective action of Msb2^*^ was not restricted to *C. albicans* because even an *E. coli* strain was rescued from histatin-5 killing (Figure 6). Thus, we conclude that protection by the secreted Msb2 glycofragment is not specific for LL-37 but extends to other AMPs including histatin-5 and affects microorganisms other than *C. albicans*.

## Discussion

**Figure 7:**
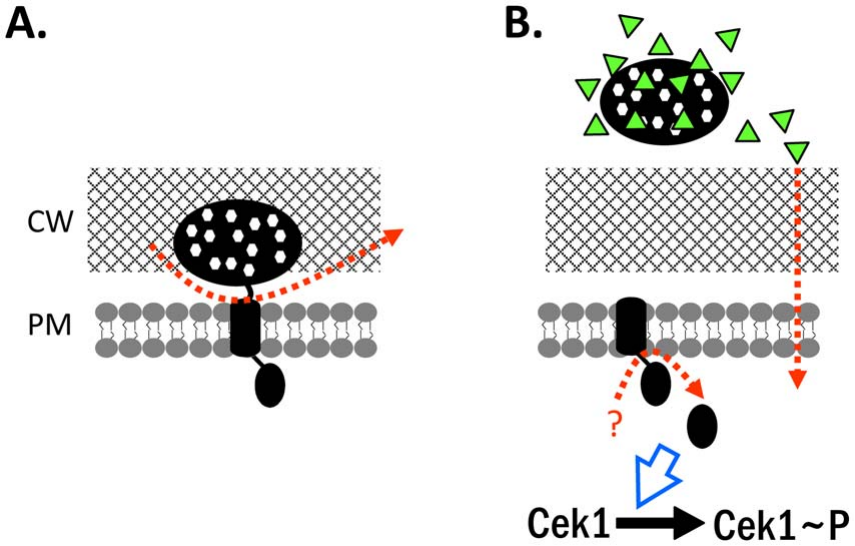
Model for Msb2 functions. The dual function of *C. albicans* Msb2 protein is shown. The precursor protein (A) is cleaved during growth and the extracellular domain, which is highly O-glycosylated (indicated by white dots), is shed into the medium (B). Msb2 has an intracellular function in activating the Cek1 MAP kinase and the secreted exodomain is able to protect cells against AMPs (triangles).

A complex interplay of responses and counter-responses characterizes the encounter of microbial pathogens with the human host. Opportunistic pathogens including *C. albicans* may be commensals, held in check by the immune system and supported by actions of the pathogen that favour a commensal life-style [1], [34]. Conversely, immunological impairment or other conditions can favour propagation of pathogens and result in disease through microbial virulence traits and/or immune hyperstimulation causing autoimmune damage [35] Immune cells detect surface structures of *C. albicans* including glucan and mannoproteins and trigger IL-17-dependent reactions [2], [3] including the production of AMPs, which kill the pathogen and attract immune cells [19], [20]. The *C. albicans* protein Msb2 has a dual function to stabilize the fungal cell wall and we show here that it is also required to block an important aspect of the immune response by inactivating AMPs (Figure 7).

Fungal pathogens have a relatively high ability to resist attack by hydrolytic enzymes or small toxic molecules including antifungals in the human host. Cell wall damage is restored or compensated for by signaling pathways that sense the defect and initiate appropriate rescue responses [6]. In *C. albicans* defects in glucan or chitin are sensed especially by pathways containing the Mkc1 or Hog1 MAP kinases that trigger enhanced glucan or chitin biosynthesis [7], [36]. Defects in protein glycosylation are transmitted mainly via the Cek1 MAP kinase pathway and lead to activation of individual isoforms of protein-*O-*mannosyltransferases [9], [10]. Blockage of *N-*glycosylation by tunicamycin depends on Cek1 and upregulates *PMT1* transcription, while inhibition of Pmt1-*O-*glycosylation stimulates transcription of *PMT2* and *PMT4* genes. Interestingly, we found that the Msb2 membrane sensor protein functioning at the head of the Cek1 pathway is itself a highly glycosylated protein as in other fungal species. Despite the presence of 5 potential acceptor sites no evidence for *N-*glycosylation of Msb2 was obtained but the secreted Msb2 migrated faster in a *pmt1* mutant (not in other homozygous *pmt* mutants) indicating that Pmt1 is partially responsible for Msb2 *O-*mannosylation. Residual *O-*chains in a *pmt1* strain were removed by chemical treatment suggesting that they are contributed by the Pmt2 isoform, which is essential for growth [27]. Lack of Pmt1 glycosylation was previously shown to increase phosphorylation of Cek1 and to activate *PMT2/4* transcription [9], [10] and we add here that lack of the N-terminal Msb2 glycodomain leads to constitutive Cek1 phosphorylation. Conceptually, lack of Msb2 *O-*glycosylation could trigger Cek1 phosphorylation but other *O-*glycosylated proteins interacting with Msb2 could also provide the triggering signal. Signaling by proteins interacting with Msb2 is suggested by the finding that tunicamycin-treatment induces Cek1 phosphorylation, although Msb2 does not appear to be *N-*glycosylated itself. In *S. cerevisiae*, however, Msb2 is *N-*glycosylated and *O-*mannosylated by the Pmt1, 2 and 4 isoforms; furthermore, activation of the Cek1 homolog Kss1 occurred only in cells lacking Pmt4 and inhibited for *N-*glycosylation by tunicamycin [37], [38]. Thus, Msb2 glycosylation and resulting MAP kinase activation proceed differently in *C. albicans* and *S. cerevisiae*.

The single transmembrane region of Msb2 divides the protein in a large glycosylated extracellular and a small cytoplasmic domain in *C. albicans*, *S. cerevisiae* and other fungi. A *S. cerevisiae* Msb2-GFP fusion has been shown to get efficiently cleaved leading to release of the extracellular domain into the medium [17]. This processing occurs at a yet undefined site and requires the Yps1 yapsin-type protease suggesting that it is directly or indirectly involved in the cleavage. Similarly, using doubly epitope-tagged Msb2 we found that in *C. albicans* Msb2 is cleaved almost quantitatively, which sheds the extracellular domain into the medium and retains the cytoplasmic domain in the cells. However, in *C. albicans* the closest homologs of ScYps1, Sap9, Sap10 [29], and serine endoproteinase Kex2 [30] were not required for CaMsb2 processing. Cleavage/release was found to occur both in liquid and on surfaces and the amount of secreted Msb2 depended on the number of growing *C. albicans* cells. Thus, importantly, the level of released Msb2 is a measure of *C. albicans* propagation. In agreement, Msb2 peptides were recently identified in the secretome of *C. albicans* yeast and hyphal cultures; peptides corresponded to the extracellular domain including residue 1290 upstream of the transmembrane region [39].

The relationship between Msb2 structure, processing/secretion and Cek1 phosphorylation was studied using *C. albicans* strains producing Msb2 variants. A large deletion of 450 N-terminal residues adjacent to the signal sequence (Msb2-ΔN) led to functional Msb2 able to complement defects of the *msb2* mutant; this variant differed from the native protein, however, in that the Cek1 MAP kinase was constitutively phosphorylated. In agreement, *S. cerevisiae* Msb2 deletions of the extracellular domain have been found to hyperactivate the dedicated MAP kinase Kss1 [17]. Different phenotypes were obtained for C-terminal deletions of *C. albicans* Msb2. While a Msb2 variant deleted for its C-terminal end and the transmembrane region (Msb2-ΔTM-C) was completely inactive, a deletion retaining the transmembrane region (Msb2-ΔC) was fully functional in complementing *msb2* phenotypes. Unexpectedly, however, the latter variant did not respond to tunicamycin-treatment by induction of Cek1 phosphorylation, in agreement with results obtained for a similar *S. cerevisiae* Msb2 variant [38]. We conclude that the transmembrane region of Msb2 is absolutely required for Msb2 functions and furthermore, that tunicamycin-regulated signaling to the Cek1 MAP kinase requires the cytoplasmic domain. Conceivably, the cytoplasmic domain could be directly involved in regulation of Cek1 kinase activity or it could participate in gene regulation as has been reported for signaling mucins and the Notch protein in higher eukaryotes [18], .

In the human host *C. albicans* contacts surfaces of body cells including immune cells, which may phagocytose the pathogen and elicit a wave of antifungal activities. Resident or induced soluble defense molecules such as immunoglobulins, complement factors and AMPs kill or block the growth of the pathogen. AMPs have a wide range of antiviral, antibacterial and antifungal activities and provide an antimicrobial barrier on mucosal surfaces such as histatins produced and secreted by salivary glands or they are components of the antimicrobial armory of neutrophils that produce cathelicidins (LL-37) and defensins [20]. Furthermore, AMPs act as chemoattractants recruiting leukocytes to sites of infection [19], [21]. *C. albicans* is known to be sensitive to histatins, LL-37 and defensins, which inhibit fungal growth by cytoplasmic membrane disruption, interference with mitochondrial activity or yet undefined mechanisms [23]–[26]. Furthermore, binding of LL-37 or histatins to cell wall carbohydrates prevents adhesion of *C. albicans* to host cells and plastic surfaces [31]. It should be noted also that bacterially-produced AMPs such as the lantibiotic nisin secreted by *Lactobacillus lactis* contribute to the diversity and high concentration of AMPs in the human body [41]. Nevertheless, a myriad of microbial commensals including some opportunistic pathogens persist as cohabitants because they are at least partially AMP-resistant. Several AMP-resistance mechanisms have been reported. Cleavage of AMPs by soluble or membrane-bound proteases has been described for many bacterial species and it has been shown that *C. albicans* is also able to cleave histatin-5 by the yapsin-type protease Sap9 [42], [43]. Another evasion mechanism known in bacteria is the secretion of AMP-binding proteins that act as decoys deflecting AMPs from their dedicated action at microbial cell surfaces. Examples include the secreted SIC, staphylokinase and FAF proteins by *Streptococcus pyogenes*, *Staphylococcus aureus* and the commensal *Finegoldia magna*, respectively [44]–[46]. Here we describe that an analogous mechanism is relevant also for fungal pathogens since shedding of a large glycosylated fragment of the Msb2 sensor protein renders *C. albicans* AMP-resistant. Msb2 shedding reached high levels during liquid growth (about 150 µg/ml in stationary phase) and was also observed during surface growth. Purified Msb2 fragment effectively blocked the fungicidal activity of histatin-5 and LL-37 even at a >20 fold molar excess of AMPs suggesting multiple binding sites. Interestingly, a *C. albicans msb2* mutant was supersensitive to LL-37 but not to histatin-5 suggesting that the relatively small amount of cell-associated Msb2 suffices to protect against LL-37 but not against histatin-5. This finding agrees with the recent finding that LL-37 but not histatin-5 binds to *C. albicans* cell-wall carbohydrates [31]. The underlying molecular mechanisms for AMP binding to Msb2* remain to be determined. We found that the Pmt1-type of *O-*mannosylation is partially required for Msb2 glycosylation, its binding to LL-37 and for LL-37 resistance of wild-type cells, which raises the question if the glycostructures of Msb2* directly or indirectly affect LL-37 binding. Previous work has established the binding of LL-37 to various glycostructures including bacterial lipopolysaccaride [47], bacterial exopolysaccharides [48], human glycosaminoglycans [49] and fungal cell-wall polysaccharides [31]. These glycostructures may provide anionic contact sites for cationic AMPs such as LL-37 and histatin-5, which are enriched for basic amino acids (net charge +6 and, respectively, +12 at physiological pH). Since *O-*mannosyl side chains of Msb2* do not add net charge (unless they carry as yet undefined modifications) they do not allow ionic interactions with cationic AMPs, although non-ionic interactions cannot be excluded. Possibly, the functional role of *O-*mannosylation is indirect by providing an extended, bottle-brush conformation of the protein, as it is often observed in highly *O-*glycosylated protein domains [50]; this conformation could help to expose carboxylate side groups of aspartate and glutamate residues in Msb2* that could interact with basic residues of AMPs. Other *C. albicans* components including members of the Hog1 MAP kinase pathway are also involved in basal AMP resistance [51]; since Msb2 is not an upstream element in the Hog1 pathway of *C. albicans* [52] it probably regulates AMP resistance independently of Hog1. In a process that is analogous to functions of Msb2, the Pra1 protein of *C. albicans* is partially shed and impairs immune responses, in this case by binding of human factor H in solution leading to downregulation of the complement system in the vicinity of fungal cells [53].

We reported previously that in the standard mouse model of systemic infection (tail vein injection) no significant attenuation of virulence was detected for a *msb2* mutant [9]. However, the systemic infection model may not appropriately reflect growth of *C. albicans* in the form of biofilms or foci of infection within organs, which are expected to be surrounded by a diffusion cloud of shed Msb2 at high levels that cause quorum resistance depending on fungal cell numbers. Shedding of Msb2 may also be important for *C. albicans* commensal growth, e. g. survival in the gut, where it is confronted with AMPs of other microbial commensals such as nisin produced by *Lactobacillus* [41]. On the other hand, shed Msb2 is able to provide cross-protection for other species as we have shown for protection of *E. coli* against LL-37 and histatin-5. Therefore, we propose that novel models for virulence and commensalism are needed to test the biological relevance of Msb2 and its shedding. Shed Msb2 may be of diagnostic value since its levels reflect fungal growth in the human host. Shed Msb2 is highly soluble and proteolytically stable because of its extensive glycosyl modifications and its presence in body fluids may be indicative of hidden localized fungal infections.

## Materials and Methods

### Strains and media

*C. albicans* strains are listed in Table 1. In *C. albicans* strain REP18 the *MSB2* ORF of both alleles is completely removed [9]; this *msb2* mutant allele is referred to as *msb2*Δ*0*. Strain FCCa27/28 contains partially deleted alleles designated *msb2*Δ*1* (encoding the 406 N-terminal residues of Msb2), which were constructed using the URA-blaster method. A 3.8 kb genomic fragment encompassing *MSB2* was PCR-amplified using primers IPF6003-*Not*I and IPF6003-*Sac*II and cloned into pUK21 (*Not*I, *Sac*II). The large *Bam*HI-*Kpn*I fragment of the resulting plasmid was ligated to the *hisG-URA3-hisG* blaster cassette of p5921 to generate pUK-6003.ko.Urab. The *Not*I-*Sac*II disruption cassette of this plasmid was used according to the standard URA blaster protocol to partially delete both *MSB2* alleles in *C. albicans* CAI4 generating FCCa27 (Ura^+^) and FCCa28 (Ura^−^). Strain FCCa28 allows integration of *MSB2* expression vectors in the *MSB2* locus by transformation with *Hpa*I-cleaved plasmid and ectopically in *LEU2* after digestion with *Eco*RV, which place *MSB2* alleles under transcriptional control of the *MSB2* and *ACT1* promoter, respectively. The disruption was verified by colony PCR using primers IPF6003-3verif/ i-p2-Ura3ver and by Southern blottings (data not shown). *E. coli* strain DH5αF′ was used for plasmid constructions and for AMP protection experiments.

**Table 1:** *C. albicans* strains.

Strain	Genotype	Reference/Source
CAF2-1	*ura3*Δ::*imm*434/*URA3*	[55]
CAI4	*ura3*Δ::*imm434*/*ura3*Δ::*imm434*	[55]
FCCa27	as CAI4 but *msb2*Δ*1*::*hisG*/*msb2*Δ*1*::*hisG*-*URA3*-*his*G	this study
FCCa28	as CAI4 but *msb2*Δ*1*::*hisG*/*msb2*Δ*1*::*hisG*	this study
REP3	*ura3*Δ::*imm434/ura3*Δ::*imm434 his1*Δ::*hisG/his1*Δ::*hisG sho1*::*FRT/sho1*::*FRT*	[9]
REP18	*ura3*Δ::*imm434/ura3*Δ::*imm434 his1*Δ::*hisG/his1*Δ::*hisG msb2*Δ*0*::*FRT/msb2*Δ*0*::*FRT*	[9]
REP21	*ura3*Δ::*imm434/ura3*Δ:*imm434 his1*Δ::*hisG/his1*Δ::*hisG sho1*::*hisG/sho1*::*hisG-URA3-hisG msb2*Δ*0*::*FRT/msb2*Δ*0*::*FRT*	[9]
CAP1-3121	as CAI4 but *pmt1*Δ::*hisG*/*pmt1*Δ::*hisG*	[27]
SPCa2	as CAP1-3121, but *ura3*Δ::*imm434*/ *URA3*	[27]
P2-22	as CAI4 but *PMT2*/*pmt2*Δ::*hisG*	[27]
CAP4-2161	as CAI4 but *pmt4*Δ::*hisG*/*pmt4*Δ::*hisG*	[27]
P5-5711	as CAI4 but *pmt5*Δ::*hisG* /*pmt5*Δ::*hisG*	[27]
CAP2-2311	as CAI4 but *pmt6*Δ::*hisG*/*pmt6*Δ::*hisG*	[27]
CNA4	as CAI4 but *kex2*::*hisG/kex2*::*hisG*	[56]
Δsap9	as CAI4 but *sap9*::*hisG/sap9*::*hisG*	[29]
Δsap10	as CAI4 but *sap10*::*hisG/sap10*::*hisG*	[29]
Δsap9 Δsap10	as CAI4 but *sap9*::*hisG/sap9*::*hisG sap10*::*hisG/sap10*::*hisG*	[29]
CIS23	as CAI4 but *PMT1/PMT1^HA^*::*SAT1*	Schmidt and Ernst, unpublished
CIS29	as CAI4 but *PMT2/PMT2^V5^*::*URA3*	Schmidt and Ernst, unpublished
ESCa3 (−1,2,3)	as FCCa28 but *LEU2/LEU2*::pES11a (*ACT1p-MSB2* ^HA-V5^)	this study
ESCa5 (−1,2,3)	as FCCa28 but *LEU2/LEU2*::pES11c (*ACT1p-MSB2* ^HA-V5 end^)	this study
ESCa7 (−1,2,3)	as FCCa28 but *LEU2/LEU2*::pDS1044-2 (*ACT1p*)	this study
ESCa8 (−1,2,3)	as FCCa28 but *LEU2/LEU2*::pES10 (*ACT1p-MSB2* ^HA^)	this study
ESCa9 (−1,2,3)	as FCCa28 but *msb2*Δ*1/msb2*Δ*1*::pES10 (*MSB2p-MSB2* ^HA^)	this study
ESCa10 (−1,2,3)	as FCCa28 but *msb2*Δ*1/msb2*Δ*1*::pES11a (*MSB2p-MSB2* ^HA-V5^)	this study
ESCa11 (−1,2,3)	as FCCa28 but *msb2*Δ*1/msb2*Δ*1*::pES11c (*MSB2p-MSB2* ^HA-V5 end^)	this study
ESCa18 (−1,2,3)	as CAP1-3121 but *LEU2/LEU2*::pES11a (*ACT1p-MSB2* ^HA-V5^)	this study
ESCa19 (−1,2,3)	as P2-22 but *LEU2/LEU2*::pES11a (*ACT1p-MSB2* ^HA-V5^)	this study
ESCa20 (−1,2,3)	as CAP4-2164 but *LEU2/LEU2*::pES11a (*ACT1p-MSB2* ^HA-V5^)	this study
ESCa21 (−1,2,3)	as P5-5744 but *LEU2/LEU2*::pES11a (*ACT1p-MSB2* ^HA-V5^)	this study
ESCa22 (−1,2,3)	as CAP2-2311 but *LEU2/LEU2*::pES11a (*ACT1p-MSB2* ^HA-V5^)	this study
ESCa25 (−1,2,3)	as FCCa28 but *LEU2/LEU2*::pES14 (*ACT1p-MSB2-*Δ*N* ^HA-V5^)	this study
ESCa26 (−1,2,3)	as CAP1-3121 but *LEU2/LEU2*::pES14 (*ACT1p-MSB2-*Δ*N* ^HA-V5^)	this study
ESCa27 (−1,2,3)	as P2-22 but *LEU2/LEU2*::pES14 (*ACT1p-MSB2-*Δ*N* ^HA-V5^)	this study
ESCa28 (−1,2,3)	as CAP4-2164 but *LEU2/LEU2*::pES14 (*ACT1p-MSB2-*Δ*N* ^HA-V5^)	this study
ESCa29 (−1,2,3)	as P5-5744 but *LEU2/LEU2*::pES14 (*ACT1p-MSB2-*Δ*N* ^HA-V5^)	this study
ESCa30 (−1,2,3)	as CAP2-2311 but *LEU2/LEU2*::pES14 (*ACT1p-MSB2-*Δ*N* ^HA-V5^)	this study
ESCa33 (−1,2,3)	as Δsap9 but *LEU2/LEU2*::pES11a (*ACT1p-MSB2* ^HA-V5^)	this study
ESCa34 (−1,2,3)	as Δsap10 but *LEU2/LEU2*::pES11a (*ACT1p-MSB2* ^HA-V5^)	this study
ESCa35 (−1,2,3)	as Δsap9 Δsap10 but *LEU2/LEU2*::pES11a (*ACT1p-MSB2* ^HA-V5^)	this study
ESCa36 (−1,2,3)	as CNA4 but *LEU2/LEU2*::pES11a (*ACT1p-MSB2* ^HA-V5^)	this study
ESCa37 (−1,2,3)	as FCCa28 but *LEU2/LEU2*::pES15 (*PCK1p-MSB2-tail*)	this study
ESCa38 (−1,2,3)	as FCCa28 but *LEU2/LEU2*::pES16 (*ACT1p-MSB2-*Δ*C* ^HA^)	this study
ESCa39 (−1,2,3)	as FCCa28 but *LEU2/LEU2*::pES17 (*ACT1p-MSB2-*Δ*TM-C* ^HA^)	this study

Strains were grown on/in standard YPD or SD media. Pmt1-inhibitor OGT2599 was resuspended in DMSO to prepare a stock solution of 10 mM [54]. Standard drop dilution tests (10 fold dilutions to 10^−5^) were used to determine sensitivity to inhibitors. Hyphal formation was induced by growth at 37°C on YPM medium containing 2% mannitol as sole carbon source or in liquid YP medium containing 10% serum [27].

### *MSB2* expression vectors

Relevant restriction site used for the construction of *MSB2* variant alleles are shown in Figure 1A. A *MSB2* allele encoding heme agglutinin (HA)-tagged Msb2 was constructed by first PCR-amplifying the 5′-end of the *MSB2* coding region using primers Msb2-ATG-*Xho*I and IPF6003-3′ (all oligonucleotides are listed in Table S1). The PCR fragment contained a novel *Xho*I site upstream of the ATG and extended to bp position 3227 of the ORF, 50 bp downstream of the *Pst*I site. The *Xho*I-*Pst*I subclone in pUC21 was mutagenized using the Quikchange kit (Stratagene) and primers HA-hin and HA-her were used to insert the sequence encoding a single HA epitope (11 amino acids) 1500 bp downstream of the ATG start codon sequence. The 3′-end of the *MSB2* ORF was then amplified by genomic PCR using primers Msb2-int2 und Msb2-Stopp-*Xho*I-*Not*I, which generated a fragment containing a *MSB2* sequence from 61 bp upstream of the *Pst*I site to the *Xho*I site downstream of the stop codon sequence that was generated in the PCR reaction. This 3′ PCR fragment was mixed with the above 5′ *Xho*I-*Pst*I fragment and the full-length modified *MSB2* allele was generated by overlap PCR using the flanking primers Msb2-ATG-*Xho*I und Msb2-Stopp-*Xho*I-*Not*I. The resulting *Xho*I fragment was cloned downstream of the *ACT1* promoter in *C. albicans* expression vector pDS1044-1 to generate plasmid pES10.

To insert the V5 epitope-encoding sequence into *MSB2* a 1037-bp region from upstream of the *Pst*I site to the middle of cytoplasmic domain sequence was PCR amplified using pES10 as template and primers PCR1 Hin und PCR1 Mitte Her, the latter primer added V5 sequences to the PCR product. In addition, a second PCR fragment (712 bp) was generated by PCR using primers PCR2 Mitte Hin (containing the V5 sequence) und PCR2 Her (downstream of the *Apa*I site in the 3′-UTR). Because both fragments contained the V5 sequence an overlap PCR using flanking primers PCR1 Hin und PCR2 Her generated a 1695 bp PCR fragment that was cut with *Nhe*I and *Apa*I and then inserted into pES10 to replace the corresponding unmodified fragment. The resulting expression plasmid encoding the *MSB2* ^HA-V5^ allele was designated pES11a. In a similar approach, an expression vector encoding a Msb2 variant carrying the V5 epitope at the C-terminal end of Msb2 was constructed using primers PCR1 Hin, PCR1 Ende Her, PCR2 Ende Hin and PCR2 Her; the resulting plasmid was designated pES11c (*MSB2* ^HA-V5 end^).

Expression vectors encoding Msb2 variants were constructed by primer-directed mutagenesis of plasmid pES11a, using the Quikchange kit (Stratagene). Plasmid pES14 encoding Msb2-ΔN lacking residues 33–481 of Msb2 was constructed using primers *Cla*1 Del1 next1/-2, plasmid ES16 encoding the Msb2-ΔC variant lacking the cytoplasmic tail of Msb2 was constructed using oligonucleotides *MSB2* Stopp nach TM Hin/-Her and plasmid ES17 encoding the Msb2-ΔTM-C variant lacking transmembrane region and cytoplasmic tail was constructed using oligonucleotides *MSB2* Stopp vor TM Hin/-Her. Plasmid ES15 encoding the Msb2-tail variant was constructed by PCR-amplification of sequences encoding the cytoplasmic tail by primers C-Tail vor/-rück and inserting it into downstream of the *PCK1* promoter in plasmid pBI-1. Plasmids were integrated into the *LEU2* locus of strain FCCa28 as described above.

### Protein methods

Strains were grown in 50 ml YPD or SD medium at 30°C to OD_600_ = 6–10 and cells were harvested by centrifugation. Cells were washed with water and resuspended in lysis buffer (50 mM HEPES/pH 7.5; 150 mM NaCl; 5 mM EDTA; 1% Triton X-100) containing protease inhibitors (Complete, Mini, Roche). Cells were broken by shaking with glass beads at 4°C for 2×10 min on a vibrax (Janke & Kunkel, 2200 rpm) or with a FastPrep homogenizer (MP Biochemicals). Cell debris and glass beads were separated from the crude cell extract by centrifugation. For immunoblottings proteins were separated by SDS-PAGE (8%, 18% or 4–20% acrylamide) and blotted to PVDF membranes. Protein standards used were the PageRuler set (Fermentas; 11–170 kDa) or the HiMark set (Invitrogen; 31–460 kDa) of proteins. Membranes were probed using rat anti-HA monoclonal antibody (1∶2000; Roche) or mouse monoclonal anti-V5 antibody (1∶2000; Serotec) and visualized using peroxidase-coupled goat anti-rat or anti-mouse antibodies (1∶10000; Thermo) and the SuperSignal West Dura chemiluminescent substrate (Pierce).

Gel filtration chromatography was done on a Superdex 200 10/300 GL column (GE healthcare) equilibrated with SD medium. Elution characteristics were established using a set of standard proteins (Sigma) containing carboanhydrase (23 kDa), BSA (66 kDa), ADH (150 kDa), β-amylase (200 kDa), apoferritin (434 kDa) and thyroglobulin (669 kDa); the void volume (V_0_) was determined using Blue dextran (2000 kDa). Protein elution volumes (V_e_) were monitored at 280 nm and fractions were collected by an ÄKTA prime plus (GE Healthcare) at a flow speed of 0.4 ml/min. To determine the molecular mass of secreted Msb2, strain ESCa3 (Msb2^HA-V5^) was grown in SD medium to OD_600_ = 10. Cells were removed by centrifugation and 500 µl of the medium was degassed, sterile-filtered and applied to the Superdex column. 200 µl fractions were collected and 20 µl per fraction were tested for the presence of Msb2^HA^ by immunoblotting. The approximate molecular mass of Msb2^HA^ was calculated from the standard protein graph using the equation y = 62258e−3,695x (x: Ve/Vo; y: molecular mass).

Deglycosylation reactions using PNGase F and α-mannosidase (jack bean) were carried out according to the instructions of the manufacturers (Roche; Sigma). To remove *O-*glycosylation the GlycoProfile β-elimination kit (Sigma) was used, either without or with pretreatment of the sample at 80°C. 200 µl of the ESCa3 growth medium was acetone-precipitated and resuspended in the same volume of water. 40 µl of the reagent mixture was added and the sample was incubated over night at 4°C. The sample was neutralized with HCl and 20 µl were analyzed by immunoblotting. The GlycoProfile IV kit (Sigma) was used to remove all forms of protein glycosylation by trifluoromethanesulfonic acid (TFMS). 1.5 ml of the growth medium of strain ESCa3 was lyophilized and 150 µl of TFMS was added and the proteins incubated at 4°C for 25 min. 4 µl of 0.2% bromophenol blue was added and neutralization by precooled pyridine (added drop-wise) was monitored by the yellowish coloring. This latter step was carried out in a bath of dry ice in ethanol. Reagents in the samples were removed by dialysis against PBS using Slide-A-Lyzer cassettes (Thermo).

The secreted Msb2^HA^ domain was purified by affinity chromatography from cultures grown in SD medium containing 2% casamino acids to an OD_600_ = 10 using a column (1 ml) containing agarose beads covalently coupled to 3.5 mg of monoclonal anti-HA high affinity antibody (Roche). The column equilibrated with buffer (20 mM Tris/HCl, pH 7.5; 0.1 M NaCl; 0.1 mM EDTA) and 50–400 ml of the culture medium containing Msb2^HA^ were loaded and the column was washed with 20 bed volumes of wash buffer (20 mM TrisHCl/pH 7.5; 0.1 M NaCl; 0.1 mM EDTA; 0,05% Tween 20). The Msb2^HA^ protein was eluted twice by 1 ml (1 mg) of HA peptide (Roche) in Tris-buffered saline.

Proteins on SDS-PAGE gels were routinely visualized by Coomassie blue or silver staining and protein concentrations were determined by the Bradford assay using a commercial assay kit (BioRad). Because of the high glycosylation status of Msb2* its concentration could not be determined reliably by any of these methods. Therefore, we developed a dot blot procedure, in which known molar concentrations of HA peptide were compared to Msb2* (or Msb2-ΔN*) signals resulting from reaction with the anti-HA antibody. Dilutions of a HA peptide solution (Roche) were spotted on an activated PDVF membrane and a dilution series of the sample containing unknown amounts of Msb2* was spotted alongside. The membrane was processed as for immunoblottings and the resulting signals were recorded using a Fujifilm LAS400 mini image analyzer and evaluated with the Fujifilm Multi Gauge program. The standard curve derived from the HA peptide were used to calculate molar amounts of the Msb2* sample.

Msb2* samples were assayed for protease contamination using the Protease Detection Kit (Jena Bioscience) that detects a wide variety of proteases, including serine proteases, cysteine proteases and acid proteases. Substrate solution (50 µl) and incubation buffer (50 µl) were mixed with 100 µl (50 µg) of Msb2* in TBS and incubated at 37°C for 16 h. 120 µl precipitation reagent was added and samples were incubated at 37°C for 30 min. Tubes were centrifuged at 12.000*× g* for 5 min and 50 µl of the supernatant was transferred to a flat bottom 96 well plate, 150 µl assay buffer was added and absorbance at 492 nm was measured using a plate spectrophotometer (Biotek).

### MAPK activation assay

Strains were grown over night to stationary phase in YPD medium and diluted into YPD medium to an OD_600_ = 0.1. Cells were grown to OD_600_ = 0.8 at 37°C and incubated further for 1 h in the presence (+) or absence (−) of tunicamycin (2 µg/ml). Immunoblots were prepared as described previously verifying equal loading by Ponceau red staining of the membranes [9]. Blots were probed with anti-phospho-p44/42 MAP kinase (Cell Signaling Technology) to detect phosphorylated Cek1 protein and ScHog1 polyclonal antibody (Santa Cruz Biotechnology) was used to detect all forms of Hog1 [9].

### Antimicrobial peptide assays

Over night cultures of *C. albicans* and *E. coli* DH5αF′ were diluted and grown in YPD at 30°C to an OD_600_ = 0.3. Cells were harvested by centrifugation and washed with and resuspended in PBS. Triplicate assays containing 5 µl cell suspension and 0–10 µg LL-37 (Sigma) or histatin-5 (AnaSpec Inc.) in a total volume 25 µl were incubated 1.5 h at 37°C, diluted 500 fold and plated on YPD. Colony forming units were determined after 2 d of growth at 30°C. The action of LL-37 on cells was visualized by fluorescence microscopy using LL-37-TAMRA (Innovagen).

To assay binding of LL-37 to Msb2* a microtiter plate assay was used. 10 µg Msb2* or Msb2-ΔN* in 200 µl PBS were allowed to bind wells of a 96 well flat bottom polystyrene plate over night at 4°C. The wells were washed three times with PBST (PBS containing 0.05% Tween 20). Then 200 µl of blocking buffer (5% w/v nonfat dry milk in PBST) was added for 2 hours at room temperature. Wells were washed three times and incubated with 5 µg LL-37 5-TAMRA for one hour. After washing three times, the fluorescence was measured on a Tecan infinite 200 plate reader (excitation 560 nm, emission wavelength 590 nm). In a competition experiment, following Msb2* binding, 3 µg LL-37 was added to wells and incubated for one hour before cells were washed and LL-37-TAMRA was added.

## Supporting Information

Figure S1 Phenotypes of *C. albicans* strains producing deleted Msb2 variants. **A.** Antifungal sensitivity. Sensitivities of strains to caspofungin (125 ng/ml) and tunicamycin (2 µg/ml) were tested by a drop dilution test on YPD agar. **B.** Hypha formation. Colonies of strains were photographed following growth for 2 d at 37°C on YPM agar. **C.** Detection of Msb2* in the growth medium. Strains were grown in YPD medium to OD_600_ = 6, centrifuged and the medium (20 µl) was analyzed by immunoblotting using rat anti-HA antibody. Strains included CAF2-1 (wt), ESCa3 (Msb2^HA-V5^), ESCa25 (Msb2-ΔN), ESCa37 (Msb2-tail), ESCa37 (Msb2-ΔC), ESCa39 (Msb2-ΔTM-C) and control strains FCCa27/28 (Msb2-Δ1) and CAP4-2164 (*pmt4*). The following *pmt* mutant strains carrying plasmid pES14 encoding the Msb2-ΔN variant were also tested by immunoblotting: ESca26 (*pmt1*), ESCa27 (*PMT2/pmt2*), ESCa28 (*pmt4*), ESCa29 (*pmt5*) and ESCa30 (*pmt6*).
(PDF)

Table S1 List of oligonucleotides.
(PDF)
