# Supplementary material for: Msb2 Shedding Protects Candida albicans against Antimicrobial Peptides
Source: PLoS Pathog. 2012 Feb 2;8(2):e1002501. doi: 10.1371/journal.ppat.1002501 (PMC3271078; doi:10.1371/journal.ppat.1002501)
Supplement: Figure S1 — Phenotypes of C. albicans strains producing deleted Msb2 variants. A. Antifungal sensitivity. Sensitivities of strains to caspofungin (125 ng/ml) and tunicamycin (2 µg/ml) were tested by a drop dilution test on YPD agar. B. Hypha formation. Colonies of strains were photographed following growth for 2 d at 37°C on YPM agar. C. Detection of Msb2* in the growth medium. Strains were grown in YPD medium to OD600 = 6, centrifuged and the medium (20 µl) was analyzed by immunoblotting using rat anti-HA antibody. Strains included CAF2-1 (wt), ESCa3 (Msb2HA-V5), ESCa25 (Msb2-ΔN), ESCa37 (Msb2-tail), ESCa37 (Msb2-ΔC), ESCa39 (Msb2-ΔTM-C) and control strains FCCa27/28 (Msb2-Δ1) and CAP4-2164 (pmt4). The following pmt mutant strains carrying plasmid pES14 encoding the Msb2-ΔN variant were also tested by immunoblotting: ESca26 (pmt1), ESCa27 (PMT2/pmt2), ESCa28 (pmt4), ESCa29 (pmt5) and ESCa30 (pmt6). (PDF) [file ppat.1002501.s001.pdf]

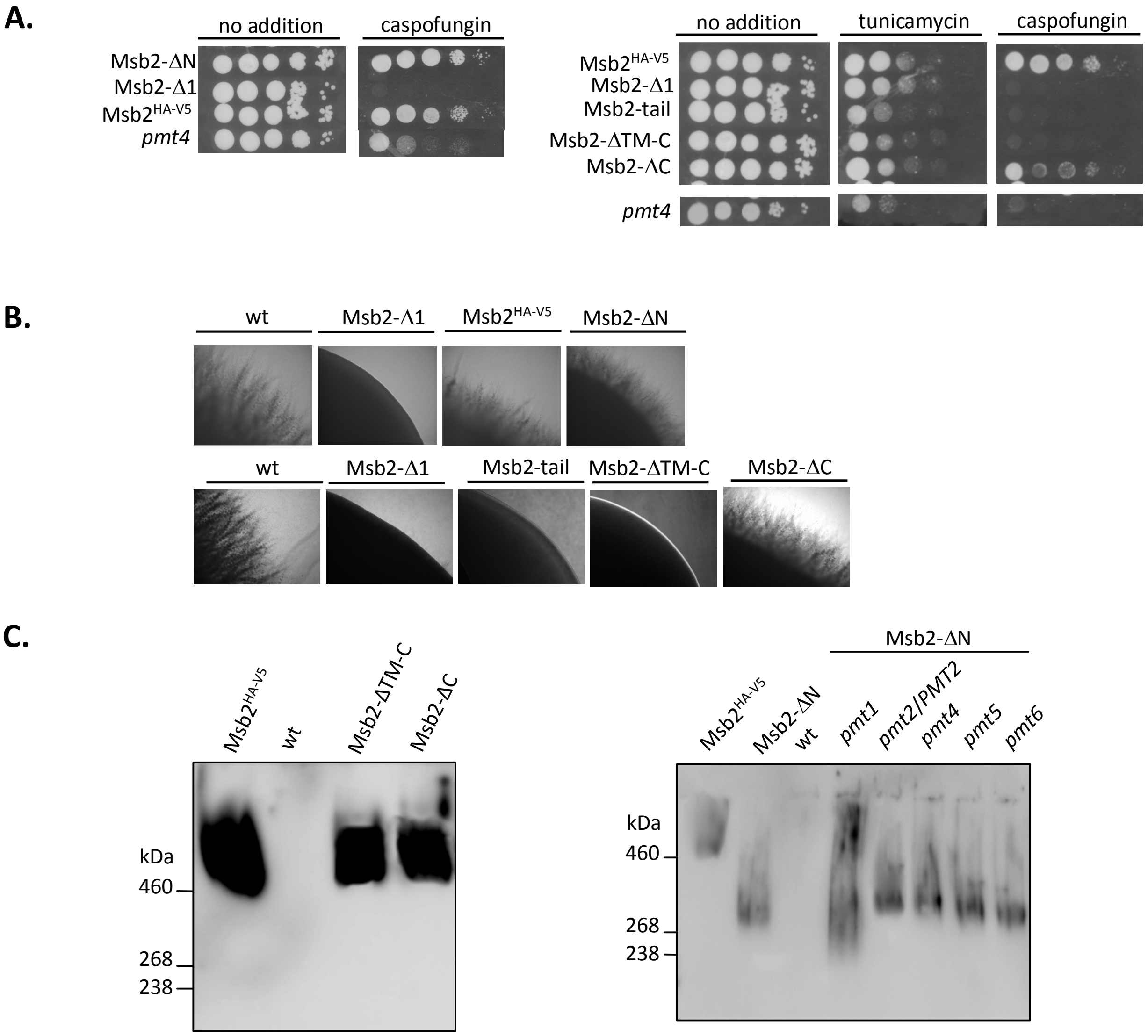

Supplemental Fig. S1. Phenotypes of *C. albicans* strains producing deleted Msb2 variants. **A.** Antifungal sensitivity. Sensitivities of strains to caspofungin (125 ng/ml) and tunicamycin (2 µg/ml) were tested by a drop dilution test on YPD agar. **B.** Hypha formation. Colonies of strains were photographed following growth for 2 d at 37 °Con YPM agar. **C.** Detection of Msb2* in the growth medium. Strains were grown in YPD medium to OD_{600}= 6, centrifuged and the medium (20 µl) was analyzed by immunoblotting using rat anti-HA antibody. Strains included CAF2-1 (wt), ESCa3 (Msb2$^{HA\text{-}V5}$), ESCa25 (Msb2-ΔN), ESCa37 (Msb2-tail), ESCa37 (Msb2-ΔC), ESCa39 (Msb2-ΔTM-C) and control strains FCCa27/28 (Msb2-Δ1) and CAP4-2164 (*pmt4*). The following *pmt* mutant strains carrying plasmid pES14 encoding the Msb2-ΔN variant were also tested by immunoblotting: ESca26 (*pmt1*), ESCa27 (*PMT2/pmt2*), ESCa28 (*pmt4*), ESCa29 (*pmt5*) and ESCa30 (*pmt6*).
