# Supplementary material for: Msb2 Shedding Protects Candida albicans against Antimicrobial Peptides
Source: PLoS Pathog. 2012 Feb 2;8(2):e1002501. doi: 10.1371/journal.ppat.1002501 (PMC3271078; doi:10.1371/journal.ppat.1002501)
Supplement: Table S1 — List of oligonucleotides. (PDF) [file ppat.1002501.s002.pdf]

Supplemental Table 1. List of oligonucleotides

name	sequence
IPF6003-NotI	5´-ATCTAGCGGCCGCGTCTATTTTGATACCCACCCC-3´
IPF6003-SacII	5´-TCAGTACCGCGGCTTGATGGCTCAGCTGATGC-3´
IPF6003-3verif	5´-CTGCTGAAGGAGCAACTGCG-3´
i-p2-Ura3ver	5´-TTACAATCAAAGGTGGTCC-3´
Msb2-ATG-*Xho*I	5´-TGAATCTCGAGATGTTGGCCAACGTTAAATTG-3´
IPF6003-3´	5´-CTTGATGGCTCAGCTGATGC-3´
HA-hin	5´-GAAACCACTTCATTAGTGAGATCTTACCCATACGATGTT CCTGACTATGCGAACCCTACCGATTCCCAAATTG-3´
HA-her	5´-CAATTTGGGAATCGGTAGGGTTCGCATAGTCAGGAACAT CGTA TGGGTAAGATCTCACTAATGAAGTGGTTTC-3´
Msb2-int2	5´-GCTACTGGTTCTCAAGTTAC-3´
Msb2-Stopp-*Xho*I-*Not*I	5´-ATTCAGCGGCCGCCTCGAGCTAATGATACCAACCCAA TG-3´
PCR1 Hin	5´-CAACAGCTGCTAGCGAG-3´
PCR1 Mitte Her	5´-CCCGGGCGTAGAATCGAGACCGAGGAGAGGGTTAGG GATAGGCTTACCACCACCTTCTAATGCCTTATTAC-3´
PCR1 Ende Her	5´-CCCGGGCTACGTAGAATCGAGACCGAGGAGAGGGTT AGGGATAGGCTTACCACCACCATGATACCAACCCAATG-3´
PCR2 Her	5´-GGGTACCGGGCCC-3´
PCR2 Mitte Hin	5´-GGTGGTGGTAAGCCTATCCCTAACCCTCTCCTCGGTC TCGATTCTACGCCCGGGTTACCAATTACTGATTC-3´
PCR2 Ende Hin	5´-GGTGGTGGTAAGCCTATCCCTAACCCTCTCCTCGGTCT CGATTCTACGTAGCCCGGGCTCGAGGCGAGTG-3´
*Cla*1 Del1 next1	5´-CCATCGATGAAATCTCTGAAAAAATTACCAATAGC-3´
*Cla*1 Del1 next2	5´-CCATCGATGATTTCTCCAATCTGGTATTGCATAGTTC-3´
C-Tail vor (*Xho*I)	5´-CCGCTCGAGATGAGAAAGTTTAGAAAGAG-3´
C-Tail rück (*Xho*I)	5´-CCGCTCGAGTAAAGTTCTCTAATGATACC-3´
MSB2 Stopp nach TM Hin	5´-GAAAGTTTAGAAAGTAGTAAGTAGCTAGCTTAAGAGTA ATAAGGCATTAG-3´
MSB2 Stopp nach TM Her	5´-CTAATGCCTTATTACTCTTAAGCTAGCTACTTACTACTT TCTAAACTTTC-3´
MSB2 Stopp vor TM Hin	5´-CGATAAAGGAAGATAGTAAGTAGCTAGCTTAAGATTGCT GGTATAAC-3´
MSB2 Stopp vor TM Her	5´-GTTATACCAGCAATCTTAAGCTAGCTACTTACTATC TTCCTTTATCG-3´
C-Tail vor (*Bam*HI)	5´-CGCGGATCCATGAGAAAGTTTAGAAAGAG-3´
C-Tail rück (*Bgl*II)	5´-GGAAGATCTTAAAGTTCTCTAATGATACC-3´
